# Applying the mixed-blessings model and labeling theory to stigma in inclusive education: An experimental study of student and trainee teachers’ perceptions of pupils with ADHD, DLD, and intellectual disability

**DOI:** 10.3389/fpsyg.2022.910702

**Published:** 2022-10-26

**Authors:** Alexander Röhm, Michelle Grengel, Michélle Möhring, Johannes Zensen-Möhring, Cosima Nellen, Matthias R. Hastall

**Affiliations:** ^1^Qualitative Research Methods and Strategic Communication for Health, Inclusion, and Participation, Department of Rehabilitation Sciences, TU Dortmund University, Dortmund, Germany; ^2^Intellectual Disabilities in Rehabilitation and Education, Department of Rehabilitation Sciences, TU Dortmund University, Dortmund, Germany

**Keywords:** stigma, inclusive education, ADHD, DLD, intellectual disability, self-efficacy, mixed-blessings model, labeling theory

## Abstract

Institutional and individual stigmatization represent major barriers that prevent children with disabilities from accessing education. It can be presumed that children with disabilities are labeled as such even in inclusive educational settings and that teachers’ attitudes toward inclusive education and children with disabilities play a crucial role in this context. Against this background, the present study aims to (a) apply and conceptualize the mixed-blessings model in the context of stigma-related reactions to children’s disability labels in inclusive education and (b) shed light on the causal attributions of teachers that underlie stigma-related attitudes toward children with various disabilities. A 3 × 2 × 2 × 2 × 2 online experiment examined the ways in which disability-specific causes and symptoms, the type of disability in question, the children’s sex, and efficacy cues regarding educational efforts affect future teachers’ attitudes toward and expectations of inclusive education as well as their social distance toward children with disabilities. The participants in this experiment were *N* = 605 German student and trainee teachers representing different types of teaching professions. A multivariate analysis of variance (MANOVA) revealed that, in particular, the cause attributed to the disability, the depicted type of disability and the probability of learning success led to changes in attitudes. Respondents’ teaching self-efficacy and their status as students or trainees emerged as moderators of the effect of pupils’ type of disability. As a result, teacher education and training as well as communication regarding pupils with disabilities require a high degree of sensitivity to disability-specific and efficacy-related cues to prevent (accidental) professional or institutional stigmatization.

## Introduction

Although inclusive education “constitutes an international policy imperative that promotes the rights of disabled children to be educated alongside their peers in mainstream classrooms” (Liasidou, 2012, p. 168), institutional and individual stigmatization remain major barriers that prevent children with disabilities from accessing education (Cooney et al., 2006; Scior et al., 2012). In the German school system, for instance, pupils with disabilities must be diagnosed and assigned special educational needs in order to receive support in accordance with their individual conditions. More than half of pupils with identified educational needs attend special education schools, which are often separated from ordinary schools in terms of both space and content, featuring different didactic concepts and curricula. Nonetheless, according to a recent report by the German *Conference of Ministers of Education* (Kultusministerkonferenz; KMK, 2022), in 2020, approximately 44.5% of such pupils attended mainstream schools with the aim to be taught alongside pupils without disabilities in an inclusive educational setting.

Accordingly, it can be presumed that children with disabilities continue to be labeled in terms of their disability, even in inclusive educational settings. Such labelling is likely to affect teachers’ reactions to them as well as their interactions with them, which can result in stigmatization (Caslin, 2021). According to Link and Phelan’s (2001) *labeling theory*, stigmatization emerges via a social process in which “elements of labeling, stereotyping, separation, status loss and discrimination co-occur in a power situation that allows them to unfold” (p. 367). In this regard, teachers’ attitudes toward inclusive education and children with disabilities play a crucial role (Avramidis and Norwich, 2002; de Boer et al., 2011; Röhm et al., 2018) and are often shaped by information regarding and attributions of causal explanations to a disability (e.g., Lebowitz et al., 2016; Zensen and Röhm, 2021). Such information can be the result of personal experiences but can also be drawn from mass media and social media sources (Röhm et al., 2018). Building on media effects research and Zillmann and Brosius’ (2000) *exemplification theory*, single-case descriptions (i.e., exemplars) such as case vignettes are known to influence recipients’ attitudes toward certain issues (e.g., inclusive education and children with disabilities; Röhm et al., 2018). Such exemplars are perceived as a typical representative of the whole group (e.g., children with disabilities in general), and attitudes toward them (e.g., social distance) are thus generalized to the whole group (Zillmann, 2006).

Following the *mixed-blessings model* (Haslam and Kvaale, 2015), which combines assumptions drawn from Weiner’s (1986) attribution theory with Gelman’s (2009) essentialism framework, biogenetic explanations, in comparison to psychosocial explanations, are believed to reduce social distance toward affected individuals, but are also assumed to increase pessimism concerning the treatability and changeability of their condition (Kvaale et al., 2013; Lebowitz et al., 2016). To date, the mixed-blessings model has been widely used to improve our understanding of mental illness stigma in the case of adults (e.g., Dittrich et al., 2021). Although the model represents a promising framework for research on stigmatization mechanisms, the reduction of stigmatization and the promotion of positive attitudes in the context of inclusive education, it has been adopted to investigate the stigmatization of children with disabilities only rarely (e.g., Zensen and Röhm, 2021). However, the question of which disability-specific causal information should be emphasized in the context of teacher education and communication regarding children with disabilities to reduce the likelihood of accidental stigmatization remains largely unanswered.

The present study aims to (a) apply and conceptualize the mixed-blessings model to stigma-related reactions to children’s disability labels in the context of inclusive education and (b) shed light on the causal attributions by teachers that underlie stigma-related attitudes toward children with various disabilities. The model’s applicability is tested by an experiment that employs single case descriptions (case vignettes) of children with different types of disabilities in the context of inclusive education. More precisely, the study examines the ways in which disability-specific causes and symptoms, the type of disability in question, the children’s sex, and efficacy cues regarding educational efforts affect student and trainee teachers’ overall attitudes toward and expectations of inclusive education and social distance toward children with disabilities.

Originally, the mixed-blessings model developed by Haslam and Kvaale (2015) postulated that information concerning the biogenetic causes of an illness contributes to either (1) an attribution of uncontrollability (e.g., disability as a consequence of fate; *cf.* Weiner, 1986; Weiner et al., 1988) or (2) perceptions of psychological essentialism, which ascribe an illness or disability to a person’s personality (*cf.* Gelman, 2009). While the former attribution is known to reduce blame and social distance toward an affected person (Weiner et al., 1988; Dijker and Koomen, 2003), the latter supposedly increases social distance as well as prognostic pessimism regarding the changeability and treatability of the disability as well as its perceived dangerousness. However, these patterns have been confirmed only partially by various studies concerning the stigma associated with mental illness, thereby highlighting the linkage between attributed uncontrollability and decreased social distance as well as between essentialist beliefs and increased prognostic pessimism (Kaushik et al., 2016; Lebowitz et al., 2016; Lebowitz and Appelbaum, 2019). For instance, Dittrich et al. (2021) did not observe significant associations among biogenetic causes (vs. psychosocial causes), essentialist beliefs, and an increase in social distance toward persons with schizophrenia. In contrast, participants who were presented with a psychosocial causal explanation for schizophrenia indicated a relation between their biogenetic causal beliefs and increased social distance, which was mediated by their essentialist beliefs. According to those authors, their “differential findings can be accounted for by the subjects’ different readiness to subscribe to biogenetic and psychosocial causal beliefs” (Dittrich et al., 2021, p. 8), thus highlighting the importance of examining the model’s implications for anti-stigma interventions in further detail.

**Figure 1:**
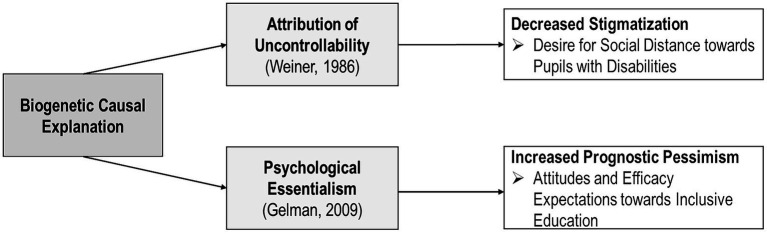
Adaptation of the mixed-blessings model to the effect of biogenetic causal explanations on stigmatization, attitudes, and expectations in the context of inclusive education.

To apply the mixed-blessings model to stigma in the context of inclusive education (Figure 1), the present study focuses on the empirically confirmed relations between biogenetic causes and, on the one hand, attributed uncontrollability (disability viewed as fate) and decreased stigmatization (i.e., social distance) as well as, on the other hand, psychological essentialism (disability viewed as an identity) and increased prognostic pessimism. *Social distance* is the most widely used operationalization of individual stigmatizing attitudes “to assess (expected) discriminatory behavior” (Baumann, 2007, p. 132). However, *prognostic pessimism* in the context of inclusive education can be understood in terms of respondents’ attitudes toward and efficacy expectations of inclusive educational efforts and settings. This approach builds on Bandura’s (1977) concept of *self-efficacy*, which is defined as a belief in one’s competence to achieve goals in a certain situation. For instance, teachers’ self-efficacy has been repeatedly linked to teaching outcomes (Klassen and Chiu, 2010) as well as their beliefs and attitudes (Dignath et al., 2022) in inclusive educational settings. Therefore, it can be presumed that descriptions of biogenetic causes can help reduce stigmatization, for example, because they lead to the belief that the affected person is not to blame for his or her condition. Simultaneously, such descriptions can also lead to the perception that the person’s condition or situation cannot be altered, for instance, by educational efforts or interventions, thereby leading to pessimistic expectations (e.g., reduced efficacy expectations). One study conducted by Zensen and Röhm (2021) examined the ways in which depictions of biogenetic, psychosocial, or bio-psychosocial explanations of ADHD in case vignettes influence student teachers’ social distance toward affected children as well as their attitudes toward inclusive education in a 3 × 2 online experiment. Their findings suggest that biogenetic causes (vs. psychosocial causes) decrease student teachers’ social distance but not their positive attitudes toward inclusive education. However, a combination of biogenetic and psychosocial causes produced the most positive attitudes. Since the study by those authors did not operationalize student teachers’ efficacy expectations of inclusive education properly, the transferability of effects on prognostic pessimism is highly limited and, hence, deserves further examination.

In light of the present research and the proposed framework, we presume that the depiction of biogenetic causes leads to less social distance (as an indicator of stigmatization) toward children with disabilities but also decreases student and trainee teachers’ positive attitudes toward and efficacy expectations (as indicators of prognostic pessimism) of inclusive education compared to cases featuring psychosocial causes:

*Hypothesis 1a*: Compared to a primary emphasis on psychosocial causes of the depicted disability, highlighting biogenetic causes reduces respondents’ social distance toward children with disabilities.

*Hypothesis 1b*: Compared to a primary emphasis on psychosocial causes of the depicted disability, highlighting biogenetic causes reduces respondents’ positive attitudes toward and efficacy expectations of inclusive educational settings.

In accordance with the assumptions of labeling theory (Link and Phelan, 2001) and *priming* (Molden, 2014), certain disability-related labels can unintentionally or unconsciously activate the stereotypical attitudes and intentions associated with that specific label. Due to the heterogeneity of pupils’ types of disability in the context of inclusive education, the present study tests the applicability of the adapted mixed-blessings model to *behavior*-related, *communication*-related, and *cognition*-related disability labels. These various labels present different challenges that pertain to teachers’ professional competencies, such as pedagogical, didactic, and educational interventions and classroom management (e.g., Blotnicky-Gallant et al., 2015). In the context of this study, *attention deficit hyperactivity disorder* (ADHD) is used as an example of a behavior-related disability label, while *developmental language disorder* (DLD) and *intellectual disability* (ID) represent communication- and cognition-related disability labels, respectively. While stigmatization of children with ID (Scior et al., 2012; e.g., Wilson and Scior, 2015; Scior and Furnham, 2016) and ADHD (e.g., Lebowitz et al., 2016; Zensen and Röhm, 2021) are well documented, little is known regarding stigma-related reactions to children with DLD (Macharey and Von Suchodoletz, 2008). Overall, intellectual disabilities are highly stigmatized due to their invisibility and the severity and controllability that are frequently attributed to them (Miller et al., 2009; Venville et al., 2016), whereas children with ADHD are perceived as noticeable and challenging but do not generally face high levels of stigmatization (Röhm et al., 2018). Regarding the stigma associated with DLD, Bishop (2017) notes that stigmatization can be associated with the specific label but also “that stigmatization is often a reaction to the child’s communication difficulties” (Bishop, 2017, p. 674).

Accordingly, it can be assumed that the depiction of a pupil with ID elicits the most stigmatization and the least efficacy expectations compared to the depictions of students with ADHD or DLD but also that a pupil with ADHD nevertheless faces more stigmatizing reactions than a pupil with DLD:

*Hypothesis 2a*: A case vignette depicting a pupil with ID is associated with greater social distance as well as fewer positive attitudes toward and efficacy expectations of inclusive education than a case vignette depicting a pupil with ADHD or DLD.

*Hypothesis 2b*: A case vignette depicting a pupil with ADHD evokes greater social distance as well as fewer positive attitudes toward and efficacy expectations of inclusive education than a case vignette depicting a pupil with DLD.

According to research findings in the context of health communication, the presentation of efficacy-related information (i.e., *efficacy cues*) influences recipients’ attitudes and behavioral intentions toward certain issues, such as vaccinations (Ort and Fahr, 2018). While teachers’ self-efficacy plays an important role in inclusive educational settings (e.g., Klassen and Chiu, 2010) and affects their general attitudes toward this topic (Savolainen et al., 2011), little is known regarding the effect of efficacy cues contained in single-case pupil descriptions on respondents’ attitudes and efficacy expectations in the context of inclusive education. In this regard, it can be assumed that the depiction of a pupil’s support needs that can be easily satisfied and offer a high chance of learning success (a *high-efficacy cue*) evoke more positive attitudes toward and higher efficacy expectations of inclusive education than the depiction of support needs that are more difficult and costly to satisfy and offer hardly any chance of learning success (a *low-efficacy cue*):

*Hypothesis 3*: Compared to a low-efficacy cue (difficult and costly support needs associated with low expectations of learning success), a high-efficacy cue (simple support needs associated with high expectations of learning success) increases respondents’ positive attitudes toward and efficacy expectations of inclusive education.

In Germany, the training of teachers is divided into two phases and differentiated in accordance with the teachers’ subsequent type of teaching profession (e.g., elementary school, secondary school, or special education). While student teachers are learning theoretical and didactic basics regarding their teaching profession while studying in bachelor’s and master’s programs at university, trainee teachers are already working in the learning environment of schools. Although both special and general education teachers are expected to work in inclusive educational settings, it is likely that general education teachers have only limited contact with students with disabilities during their training. In both parts of their training, prospective special education teachers encounter content that is specifically adapted to the target group of students with different types of disabilities. In the practical sections of the training, such teachers are required to gain experience with this special target group. Therefore, the preconditions, previous experiences, and efficacy expectations associated with teaching pupils with disabilities are expected to differ between special education and general education teachers as well as between student teachers and trainee teachers. This assumption is supported by the findings of one recent literature review of 71 studies conducted by Wray et al. (2022), who identified teacher education and training as well as teachers’ experiences with people with disability as important factors with respect to teachers’ self-efficacy in the context of inclusive education.

In light of the goals of the present study, the connection between student and trainee teachers’ general confidence in their teaching skills (i.e., their teaching self-efficacy; Savolainen et al., 2011) and their attitudes toward and efficacy expectations of inclusive education in particular deserves further attention. In this regard, Röhm et al. (2018) observed that student teachers with high teaching self-efficacy report more favorable attitudes overall toward children with disabilities than do student teachers with low teaching self-efficacy. Furthermore, teaching self-efficacy served as a moderator in a complex pattern of interaction that involves pupils’ grades and respondents’ sex, whereas “especially bad-graded pupils seem to evoke more positive attitudes in male student teachers than female student teachers with high teaching self-efficacy” (Röhm et al., 2018, 52). Thus, it can be assumed that respondents’ teaching self-efficacy affects their attitudes toward and efficacy expectations of inclusive education as well as their social distance toward children with disabilities in general. In addition, student and trainee teachers with high teaching self-efficacy are presumably more positively disposed toward inclusive education and children with disabilities when they are presented with low-efficacy cues (difficult and costly support needs associated with low expectations of learning success) than are teachers with low teaching self-efficacy. The following hypotheses are thus proposed:

*Hypothesis 4*: Student and trainee teachers with high teaching self-efficacy report more positive attitudes toward and higher efficacy expectations of inclusive education as well as less social distance than student and trainee teachers with low teaching self-efficacy.

*Hypothesis 5*: When low-efficacy cues become salient, student and trainee teachers with high teaching self-efficacy report more positive attitudes toward and higher efficacy expectations of inclusive education as well as less social distance than student and trainee teachers with low teaching self-efficacy.

Although some evidence suggests that female pupils are perceived and evaluated more positively than male pupils (e.g., Steinmayr and Spinath, 2008; Burusic et al., 2012), another study (Röhm et al., 2018) reports that student teachers indicate more stigmatization of and fewer positive attitudes toward female pupils than male pupils. Thus, the role of the sex of the depicted pupil in the process of stigmatization is addressed in the form of the following research question:

*Research Question 1*: How does the sex of the depicted pupil affect respondents’ social distance as well as their attitudes toward and efficacy expectations of inclusive education?

## Materials and methods

### Design and procedure

In a 3 × 2 × 2 × 2 × 2 online experiment, student and trainee teachers were recruited via social media and mailing lists. Each respondent was randomly assigned to one of 48 online survey questionnaires featuring a fictional case vignette depicting a pupil in an inclusive elementary school. The vignettes were experimentally manipulated regarding the pupil’s *type of disability* (ADHD vs. DLD vs. ID), *sex* (male vs. female), the *attributed cause* of the disability (biogenetic vs. psychosocial), the pupil’s *need for educational support* (low vs. high) and the pupil’s *chance of learning success* (low vs. high). Before reading the case vignette, respondents’ teaching self-efficacy in terms of a trait was assessed as a potential moderator. Subsequently, their attitudes toward and efficacy expectations of inclusive education as well as their social distance toward children with disabilities were measured as primary dependent variables. Finally, a brief manipulation check was conducted, sociodemographic data were collected, and respondents were thanked for their participation. Participants’ consent for and agreement with data collection and processing was obtained at the beginning of the survey by active confirmation in accordance with the EU General Data Protection Regulation (GDPR). All participants were informed of the context of the study prior to participating and were subsequently debriefed in detail regarding the purpose of the experimental stimulus and the details of the questionnaire. A required sample size of approximately *n* = 632 for the experiment was estimated via an a priori power analysis using G*Power (Faul et al., 2009) to conduct a multivariate analysis of variance (MANOVA) with *f*^2^ = 0.01, *p* < 0.05, and a power of 0.80.

### Sample

In total, 1,471 student and trainee teachers from various German universities were recruited for the study, of whom *N* = 605 (*M* = 25.36 years; *SD* = 5.15; 96.2% female) completed the full survey (59% dropout). Table 1 shows the distribution of the final sample by student or trainee teacher status and type of teaching profession.

**Table 1:** Sample distribution by student or trainee teacher status and type of teaching profession.

	Student teachers	Trainee teachers	Total
Type of teaching profession			
Elementary school	206	97	303
Secondary school	105	74	179
Vocational school	17	8	25
Special education	68	30	98
Total	396	209	605

### Stimulus material

**Figure 2:**
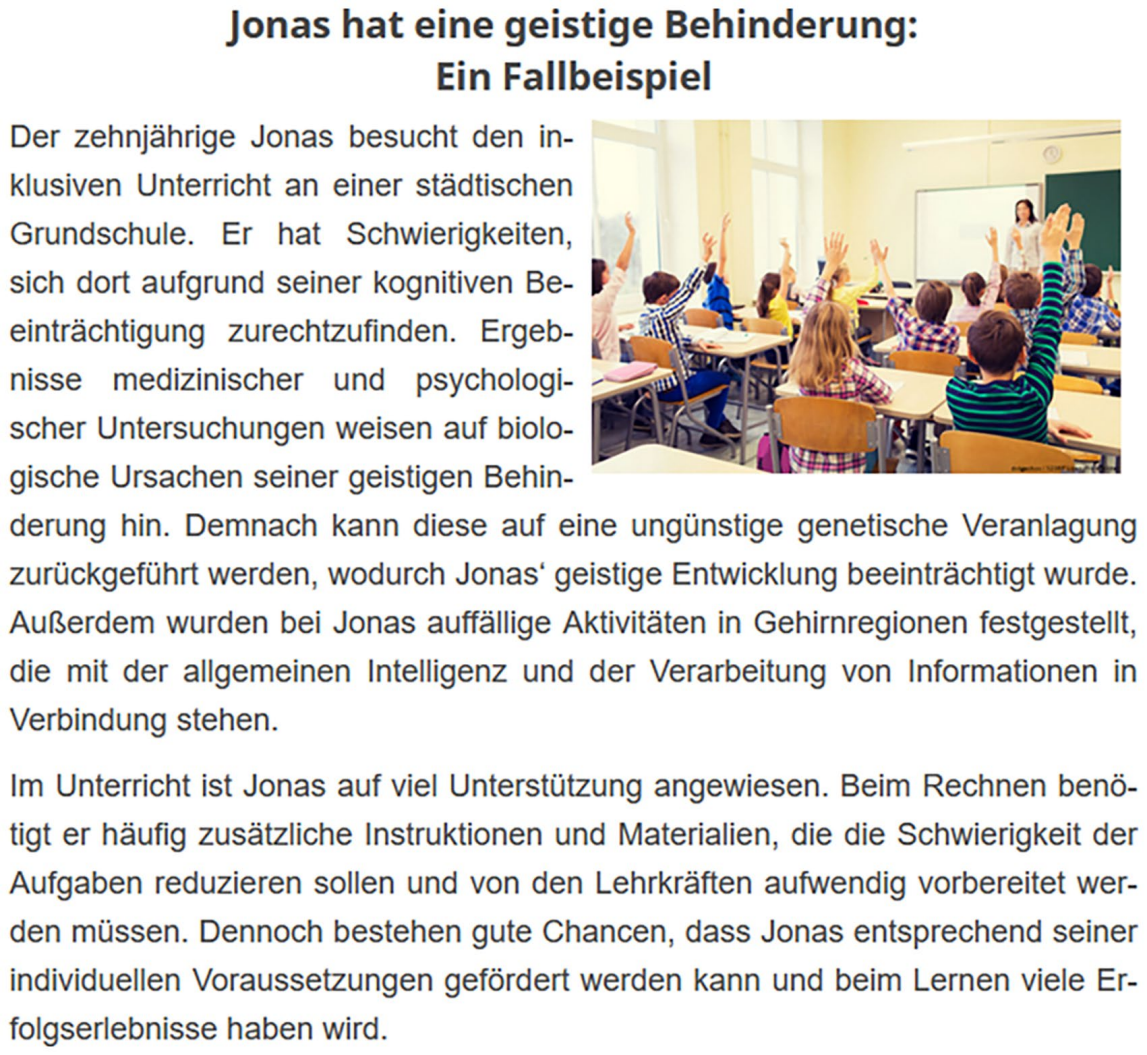
Example of stimulus material (see Table 2 for the English translation; manipulations: type of disability: intellectual disability; sex: male; attributed cause: biological; need for support: high; chance of learning success: high; Image Source: dolgachov/123rf.com).

The stimulus material consisted of a case vignette depicting a pupil in an elementary school featuring an inclusive educational setting (Figure 2; see Table 2 for the English translation). All 48 case vignettes were nearly equal in length, with *M* = 129.79 words (*SD* = 1.00). Each case vignette was illustrated using a neutral image of a class room situation, which was kept constant throughout all experimental conditions.

**Table 2:** Original German stimulus text from Figure 2 and its English translation (manipulations: type of disability: intellectual disability; sex: male; attributed cause: biological; need for support: high; chance of learning success: high).

Original German stimulus text	English translation
Jonas hat eine geistige Behinderung: Ein Fallbeispiel Der zehnjährige Jonas besucht den inklusiven Unterricht an einer städtischen Grundschule. Er hat Schwierigkeiten, sich dort aufgrund seiner kognitiven Beeinträchtigung zurechtzufinden. Ergebnisse medizinischer und psychologischer Untersuchungen weisen auf biologische Ursachen seiner geistigen Behinderung hin. Demnach kann diese auf eine ungünstige genetische Veranlagung zurückgeführt werden, wodurch Jonas’ geistige Entwicklung beeinträchtigt wurde. Außerdem wurden bei Jonas auffällige Aktivitäten in Gehirnregionen festgestellt, die mit der allgemeinen Intelligenz und der Verarbeitung von Informationen in Verbindung stehen. Im Unterricht ist Jonas auf viel Unterstützung angewiesen. Beim Rechnen benötigt er häufig zusätzliche Instruktionen und Materialien, die die Schwierigkeit der Aufgaben reduzieren sollen und von den Lehrkräften aufwendig vorbereitet werden müssen. Dennoch bestehen gute Chancen, dass Jonas entsprechend seiner individuellen Voraussetzungen gefördert werden kann und beim Lernen viele Erfolgserlebnisse haben wird.	Jonas has an Intellectual Disability: A Case Study Ten-year-old Jonas attends inclusive classes at a city elementary school. He has difficulties finding his way around there due to his cognitive impairment. The results of medical and psychological examinations point to biological causes of his intellectual disability. These causes can be attributed to an unfavorable genetic predisposition, which has impaired Jonas’ mental development. In addition, Jonas has been found to have abnormal activity in brain regions associated with general intelligence and information processing. In class, Jonas relies on a lot of support. When doing arithmetic, he often needs additional instructions and materials to reduce the difficulty of the tasks, which require extensive preparation by the teachers. Nevertheless, there is a good chance that Jonas can be supported according to his individual needs and experience a great deal of success in learning.

#### Experimental manipulations

The pupil’s type of disability is labeled ADHD to represent a behavioral disorder, DLD to indicate a communicative disorder, or ID to suggest a cognitive disorder. The pupil’s sex is indicated by the name given (male: Jonas; female: Julia) and the use of corresponding pronouns. A genetic predisposition (biogenetic) or a conflict-ridden parental home (psychosocial) are mentioned as the attributed cause of the pupil’s disability, which affects the pupil’s socioemotional, language, or cognitive development. Symptoms in all conditions are described in terms of noticeable activity in specific brain areas that are responsible for directing and focusing attention (ADHD), language acquisition and processing (DLD), or general intelligence and information processing (ID). The pupil’s need for support is illustrated by way of example in the context of arithmetic: he or she requires either a great deal of or little support and either frequent or infrequent additional instructions and materials with respect to structuring (ADHD), visualizing (DLD), or reducing the difficulty of (ID) school tasks. These instructions and materials can be prepared through the expenditure of either low (“quickly”) or high (“time-consuming”) effort by the teacher. The vignette concludes by highlighting the pupil’s prospects of learning success, indicating either a chance for a great deal of success or the risk of hardly any success.

#### Manipulation check

To estimate the correct recognition of all experimental stimulus manipulations, the respondents were asked whether they remembered (1) which type of disability (“ADHD,” “DLD,” or “ID”), (2) which sex (“male” or “female”), (3) which cause of disability (“biogenetic” or “psychosocial”), (4) which level of need for educational support (“high and costly” or “low and uncomplicated”), and (5) which chance of learning success (“hardly any chance of success” or “large chance of success”) were depicted in the case vignette. Chi-squared tests for each pair of respondents’ categorical answers and the corresponding stimulus manipulation showed that all experimental conditions were successfully and unanimously recognized (type of disability: *Χ^2^*(4, *N* = 599) = 1052.01, *p* < 0.001; sex: *Χ^2^*(1, *N* = 602) = 594.04, *p* < 0.001; attributed cause: *Χ^2^*(1, *N* = 602) = 454.54, *p* < 0.001; need for educational support: *Χ^2^*(1, *N* = 599) = 375.76, *p* < 0.001; chance of learning success: *Χ^2^*(1, *N* = 598) = 237.21, *p* < 0.001).

### Instruments

The following section describes the instruments used in this study. The reliability of each instrument is indicated by both Cronbach’s alpha and McDonald’s omega, including a standard error and 95% confidence interval based on 1,000 bootstrap samples using Hayes and Coutts (2020) OMEGA macro for SPSS.

#### Moderator: Teaching self-efficacy as a trait

Teaching self-efficacy as a trait was assessed using the *teacher self-efficacy* scale developed by Schwarzer and Schmitz (1999). This scale measures teachers’ expectations of their ability to cope with specific teaching-related situations against the backdrop of their perceived competencies and personality as a teacher based on Bandura’s (1977) social cognitive theory. Respondents were asked to indicate their levels of agreement with ten statements pertaining to an example situation (e.g., “I know that I am able to teach test-relevant content even to problematic pupils”) on a four-point Likert-type scale (0 = “not true”; 3 = “precisely true”). High scores indicate high levels of teaching self-efficacy. The scale’s reliability reached a sufficient Cronbach’s alpha of 0.744 (*SE* = 0.017; 95% BaCI[0.707; 0.775]) and McDonald’s omega of 0.742 (*SE* = 0.018; 95% BaCI[0.701; 0.775]). To facilitate the inclusion of these scores in the main analysis in the form of a categorical group variable, mean scores were calculated and dummy-coded as either 1 = ‘low teaching self-efficacy’ (*n* = 314) or 2 = ‘high teaching self-efficacy’ (*n* = 291) as determined by a median split at *Md* = 2.0.

#### Dependent variables

##### Attitudes toward and efficacy expectations of inclusive education

To operationalize prognostic pessimism in the context of inclusive education, respondents’ attitudes toward and efficacy expectations regarding that topic were assessed using the *perceived self-efficacy in inclusive education* (four items; Cronbach’s alpha = 0.811, *SE* = 0.013, 95% BaCI[0.783; 0.834]; McDonald’s omega = 0.816, *SE* = 0.013, 95% BaCI[0.789; 0.840]) and *arrangement of inclusive education* (four items; Cronbach’s alpha = 0.743, *SE* = 0.019, 95% BaCI[0.700; 0.776]; McDonald’s omega = 0.728, *SE* = 0.023, 95% BaCI[0.678; 0.769]) subscales of Bosse and Spörer’s (2014) *short scales for inclusive attitudes and self-efficacy of teachers*. Participants reported the extent to which they agreed or disagreed with assertions such as “I have the confidence to organize lessons in such a way that children like [Jonas/Julia] can achieve their goals at their own learning pace” (perceived self-efficacy) and “Joint teaching of children with and without disabilities can meet the needs of all children through appropriate methods” (arrangement of inclusive education) on a four-point Likert-type scale (1 = “fully reject”; 4 = “fully agree”). High scores indicate a high perception of self-efficacy and confidence regarding the arrangement of inclusive education, thus representing low levels of prognostic pessimism regarding the assumptions drawn from the mixed-blessings model.

##### Social distance toward children with a disability

Respondents’ tendency to distance themselves from children with a disability was assessed using eight items taken from the *social distance* subscale of the German adaptation of the *Mental Retardation Attitude Inventory* (MRAI-d; Schabmann and Kreuz, 1999). Participants indicated their levels of agreement or disagreement with statements such as “I would rather not invite a child with a disability to play with the friends of my child who do not have a disability” on a four-point Likert-type scale (1 = “do not agree at all”; 4 = “strongly agree”; Cronbach’s alpha = 0.626, *SE* = 0.072, 95% BaCI[0.444; 0.734]; McDonald’s omega = 0.615, *SE* = 0.070, 95% BaCI[0.459; 0.729]). High scores indicate a high tendency to engage in social distancing behavior.

### Instrument descriptive statistics and missing value analysis

Table 3 shows the means and standard deviations of as well as the intercorrelations among the moderator and the three dependent variables included in this study. A missing value analysis (MVA) indicated that missing values did not occur at random using Little’s MCAR (missing completely at random) test: *Χ*^2^ = 1466.22*, df* = 692, *p* < 0.001. Of the total dropout of *n* = 866 participants 45% (*n* = 395) aborted the survey before or during answering the moderator scale (i.e., teaching self-efficacy) and another 30% (*n* = 260) before the stimulus presentation. Further 22% (*n* = 185) of the participants dropped out before or during answering the dependent measures, whereas only 3% (*n* = 26) of the dropped-out participants incompletely answered one or two dependent measures and did not give any demographic information. All data analyses were conducted using IBM SPSS Version 26.

**Table 3:** Means, standard deviations, and intercorrelations of the moderator and the three dependent variables.

		*M*	*SD*	(2)	(3)	(4)
*Moderator*						
(1)	Teaching self-efficacy	2.04	0.35	0.52**	0.23**	−0.14**
*Dependent variables*						
(2)	Perceived self-efficacy	2.80	0.63		0.39**	−0.08*
(3)	Arrangement of inclusive education	3.19	0.62			−0.15**
(4)	Social distance	1.07	0.19			

** *p* < 0.01;

* *p* < 0.05.

## Results

All hypotheses and the research question were tested by conducting a MANOVA including the five experimental manipulations (1. type of disability; 2. sex; 3. attributed cause of disability; 4. need for educational support; 5. chance of learning success) as well as respondents’ teaching self-efficacy (median split) and student/trainee status as factors for all three dependent variables. Respondents’ sex was not included as a factor to prevent unequal distributions among the experimental and quasi-experimental factors. To ensure sufficient cell sizes with *n* > 30 respondents per cell, the MANOVA model was limited to main effects, two-way interactions, and three-way interactions. Concerning potential statistical outliers, the calculation of Mahalanobis distances yielded *n* = 11 cases (1.8%) above the chi-squared distribution cutoff value of 16.266 (*df* = 3, *p* < 0.001). Due to the comparably low number of outliers and the general robustness of MANOVAs against extreme values (*cf.* Field, 2018), these cases were included in the analyses. To protect subsequent univariate analyses of variance (ANOVAs) against type I error, only effects of the MANOVA with *p* ≤ 0.01 are reported (Field, 2018). Table 4 displays all significant main effects and higher-order interactions uncovered by the MANOVA using Pillai’s trace. The significant main effects and higher-order interactions that emerged from subsequent ANOVAs are reported below. Effect sizes are indicated by partial eta-squared ($\eta_{p}^{2}$) for subsequent ANOVAs based on the output of SPSS (*cf.* Ellis, 2010; Lakens, 2013; Field, 2018). The significance of differences between the estimated marginal means was determined using Sidak-corrected simple effect post hoc tests.

**Table 4:** Significant main effects and higher order interactions shown by the MANOVA regarding all five experimental manipulations alongside respondents’ teaching self-efficacy and student or trainee status as factors using Pillai’s trace.

	*V*	*F*	*df1*	*df2*	*p*
*Main effects*					
Attributed cause of disability	0.028	4.986	3	517	=0.002
Respondents’ teaching self-efficacy	0.188	39.816	3	517	<0.001
*Higher-order interaction of stimulus manipulations*					
Type of disability × Chance of learning success	0.032	2.852	6	1,036	=0.009
*Higher-order interaction of stimulus manipulations and respondents’ characteristics*					
Type of disability × Respondents’ teaching self-efficacy × Respondents’ student/trainee status	0.032	2.803	6	1,036	=0.010

Perceived self-efficacy in inclusive education, arrangement of inclusive education, and social distance were included as dependent variables.

### Main effect of the cause attributed to the disability

A main effect of the cause attributed to the disability became significant with respect to arrangement of inclusive education, *F*(1,519) = 12.547, *p* < 0.001, $\eta_{p}^{2}$ = 0.024. Compared to the depiction of a psychosocial cause for the disability (*M* = 3.27; *SE* = 0.04), the depiction of a biogenetic cause led to significantly fewer positive attitudes regarding the arrangement of inclusive education (*M* = 3.07; *SE* = 0.04; *p* < 0.001), a result which indicates higher prognostic pessimism, thus supporting Hypothesis 1b.

### Main effects of respondents’ teaching self-efficacy

Significant main effects of respondents’ teaching self-efficacy emerged with respect to perceived self-efficacy in inclusive education, *F*(1,519) = 117.986, *p* < 0.001, $\eta_{p}^{2}$ = 0.185, and arrangement of inclusive education, *F*(1,519) = 20.079, *p* < 0.001, $\eta_{p}^{2}$ = 0.037. Respondents in the group with high teaching self-efficacy indicated significantly more perceived self-efficacy in inclusive education (*M* = 3.08; *SE* = 0.04) and confidence in the arrangement of inclusive education (*M* = 3.30; *SE* = 0.04) after reading the case vignette than respondents with low teaching self-efficacy (perceived self-efficacy: *M* = 2.51, *SE* = 0.04, *p* < 0.001; arrangement of inclusive education: *M* = 3.05; *SE* = 0.04; *p* < 0.001). These findings support Hypothesis 4.

### Higher-order interaction of stimulus manipulations

**Figure 3:**
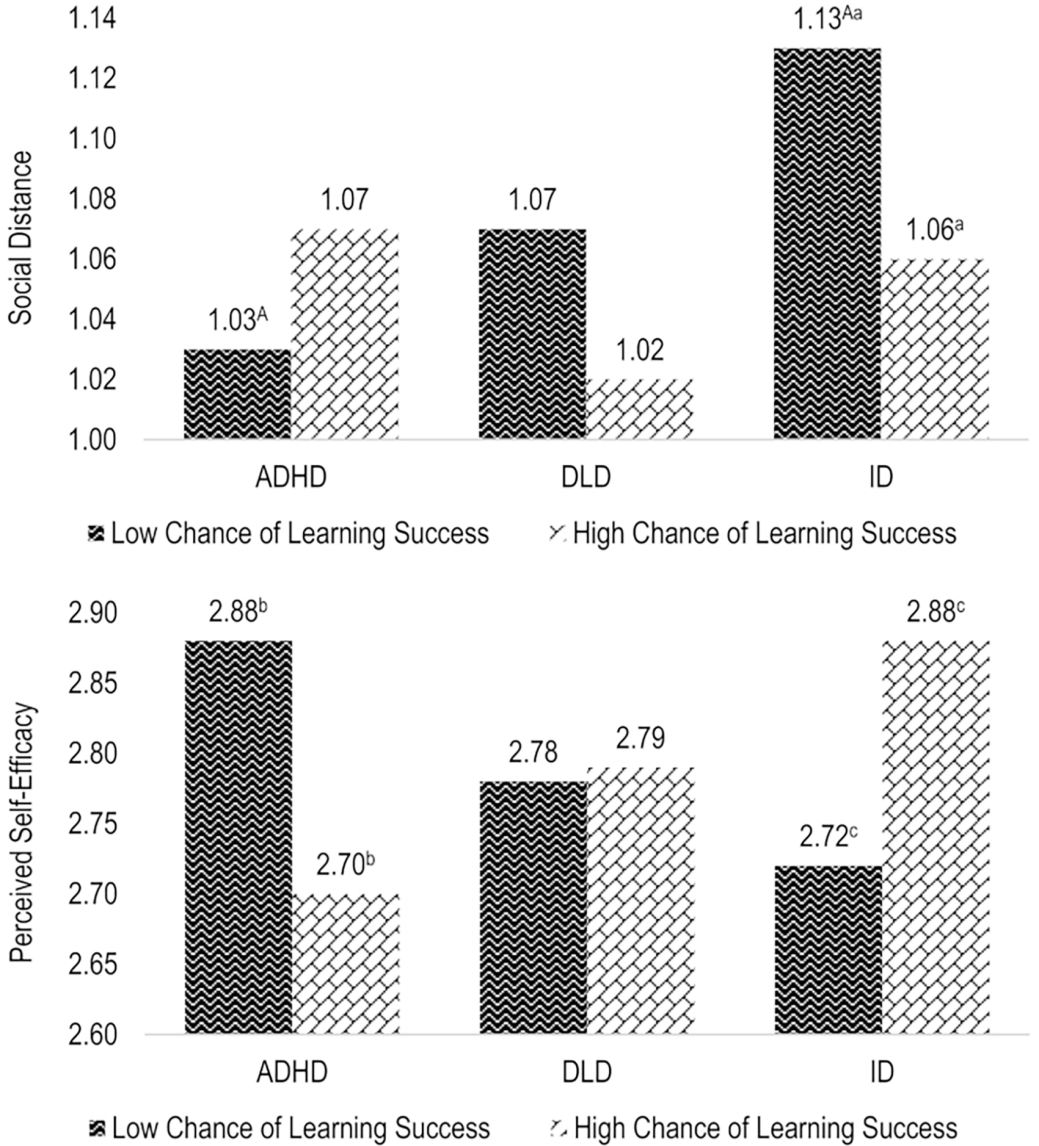
Estimated marginal means of the effect of the two-way interaction between type of disability × chance of learning success on social distance and perceived self-efficacy in the context of inclusive education. Means sharing the same capital letter differ significantly at *p* < 0.01. Means sharing the same lower case letter differ significantly at *p* < 0.05 (Sidak-corrected post hoc comparisons).

A significant two way interaction between type of disability × chance of learning success appeared with respect to social distance, *F*(2,519) = 3.922, *p* = 0.020, $\eta_{p}^{2}$ = 0.015, and perceived self-efficacy in inclusive education, *F*(2,519) = 3.809, *p* = 0.023, $\eta_{p}^{2}$ = 0.014. Case vignettes depicting a pupil with ID evoked significantly greater social distance than case vignettes depicting a pupil with ADHD when the ascribed chance of learning success was low (Figure 3). In addition, the depiction of a pupil with ID and a low chance of learning success led to greater social distance and lower perceived self-efficacy than the depiction of a pupil with the same disability and a high chance of learning success. In contrast, depicting a pupil with ADHD and a low chance of learning success led to higher perceived self-efficacy than depicting a pupil with the same disability and a high chance of learning success.

### Higher-order interaction of stimulus manipulation and respondents’ characteristics

**Figure 4:**
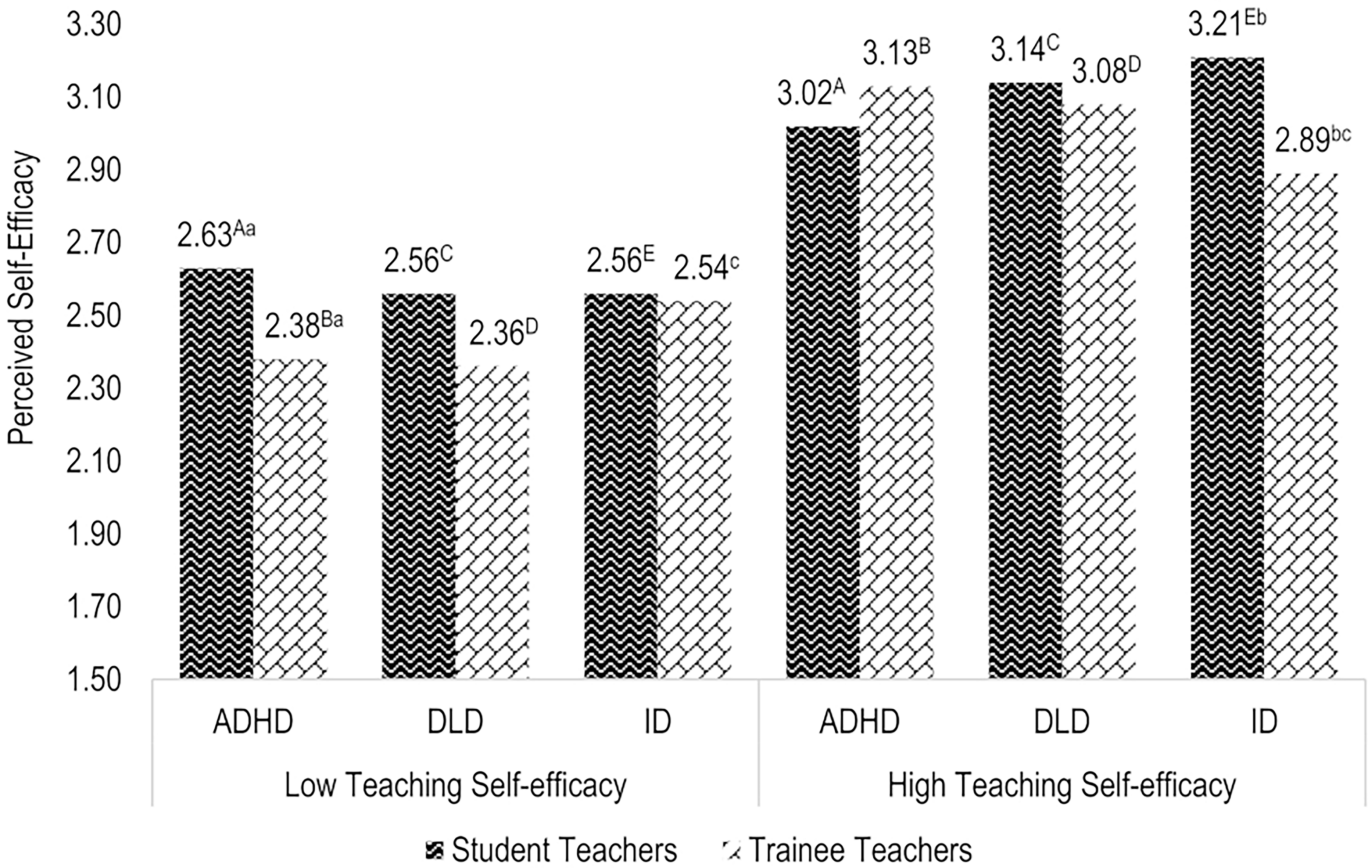
Estimated marginal means of the effect of the three-way interaction between type of disability × respondents’ teaching efficacy × respondents’ student/trainee teacher status on perceived self-efficacy in the context of inclusive education. Means sharing the same capital letter differ significantly at *p* < 0.01. Means sharing the same lower case letter differ significantly at *p* < 0.05 (Sidak-corrected post hoc comparisons).

The three way interaction among type of disability × respondents’ teaching self-efficacy × respondents’ student/trainee status became significant with respect to perceived self-efficacy, *F*(2,519) = 3.600, *p* = 0.028, $\eta_{p}^{2}$ = 0.014. Figure 4 indicates that trainee teachers with low teaching self-efficacy reported significantly less perceived self-efficacy after reading a case vignette featuring a pupil with ADHD than did student teachers with low teaching self-efficacy. Likewise, trainee teachers with high teaching self-efficacy reported significantly less perceived self-efficacy after reading a case vignette featuring a pupil with ID than did student teachers with high teaching self-efficacy. Moreover, both student and trainee teachers with low teaching self-efficacy indicated significantly less perceived self-efficacy in the context of inclusive education in all disability conditions than did student and trainee teachers with high teaching self-efficacy.

## Discussion

The aim of the present study was to shed light on student and trainee teachers’ perceptions of pupils with disabilities in the context of inclusive education. In accordance with assumptions drawn from exemplification theory (Zillmann and Brosius, 2000), respondents’ attitudes and expectations regarding this topic were generally influenced by the single-case depictions (i.e., exemplars) that were presented. In particular, the cause attributed to the disability, the depicted type of disability and the chance of learning success led to attitude changes. Respondents’ teaching self-efficacy and status as students or trainees emerged as moderators in this regard.

Specifically, the observed effect of an attributed biogenetic cause of the pupil’s disability (vs. a psychosocial cause) on decreased positive attitudes regarding the arrangement of inclusive education supports Hypothesis 1b (i.e., that highlighting biogenetic causes reduces respondents’ positive attitudes and efficacy expectations compared to cases highlighting psychosocial causes). In line with the assumptions drawn from the mixed-blessings model (Haslam and Kvaale, 2015), emphasizing a biogenetic cause decreased respondents’ beliefs regarding the successful design of inclusive education settings for children with the depicted disability, thus indicating increased prognostic pessimism regarding this topic. With respect to the model’s underlying assumptions in terms of psychological essentialism (Gelman, 2009), this finding could indicate that a case vignette featuring a biogenetic cause would lead to a perception of the depicted disability as part of the pupil’s personality, which would make it unresponsive to educational efforts. However, since this manipulation had no significant effects on either perceived self-efficacy in inclusive education or social distance, no further support was provided for Hypothesis 1b, and no support at all was found for Hypothesis 1a (i.e., that highlighting biogenetic causes reduces respondents’ social distance toward children with disabilities compared to cases highlighting psychosocial causes). Thus, the adoption of the mixed-blessings model to stigma in the context of inclusive education is only partially supported by the present results. Moreover, no interactions between the depicted cause and the pupil’s type of disability were found, indicating a lack of disability-specific effects. In contrast to previous findings (Lebowitz et al., 2016; Zensen and Röhm, 2021), it was not the case for any of the three disability labels (ADHD, DLD, or ID) that a suggested biogenetic cause evoked an attribution of uncontrollability resulting in reduced social distance toward affected children. Since intellectual disabilities are generally highly stigmatized due to their alleged severity and controllability (e.g., Venville et al., 2016), it is possible that teachers’ perceptions of the controllability or uncontrollability of the pupils’ ID became equalized between the biogenetic and the psychosocial condition. Dittrich et al. (2021) describe a similar effect found by their test of the mixed-blessings model regarding persons with schizophrenia. Accordingly, respondents may have been unready to change their original attributions on the basis of the mere presentation of the challenging biogenetic causal explanation. Concerning depictions of pupils with DLD, little is known regarding attributions of controllability. Thus, emphasizing neither a biogenetic nor a psychosocial cause made a difference in terms of the stigmatization of pupils with DLD in our study. It remains unclear, however, why an attributed biogenetic cause did not lead to any differences in respondents’ social distance toward affected children in the case of the vignettes featuring a pupil with ADHD.

Although no sole main effects of type of disability emerged, the observed interaction effects with the depicted chance of learning success provide some insights into the ways in which stigmatizing attitudes regarding specific disability labels can be shaped by the prospect that educational efforts will have a positive outcome. In this regard, pupils with ID were most stigmatized and associated with the lowest self-efficacy expectations when their chance of learning success was described as low, a finding which is to some extent in line with Hypothesis 2a (i.e., that a pupil with ID is associated with greater social distance as well as fewer positive attitudes and efficacy expectations than a pupil with ADHD or DLD). However, when the pupil’s chances of learning success were high, the ID label did not elicit more stigmatization than ADHD or DLD. Moreover, respondents’ perceived self-efficacy regarding a pupil with ID and a high chance of learning success was as high as that concerning a pupil with ADHD and a low chance of learning success. While there were no significant differences whatsoever regarding pupils with DLD and, therefore, no support for Hypothesis 2b (i.e., a pupil with ADHD evokes greater social distance as well as fewer positive attitudes and efficacy expectations than a pupil with DLD), the prospect of a positive or negative learning outcome for pupils with an ADHD label seemed to alter respondents’ reactions in the opposite direction compared to cases of an ID label. Case vignettes featuring a pupil with ADHD and a low chance of learning success evoked the least stigmatization and the most perceived self-efficacy in the context of inclusive education, whereas the combination of an ADHD label and a high chance of learning success resulted in the lowest efficacy expectations. A possible explanation for such a counterintuitive effect pattern could be that, on the one hand, the high degree of stigmatization associated with a low-achieving pupil with ID highlights respondents’ general expectations regarding children with ID and reflects the public stigma of individuals with ID (Venville et al., 2016). The prospect of a positive outcome, in contrast, provides an effective efficacy cue that children with ID can benefit from inclusive educational efforts. Accordingly, the reduction in stigma and increase in efficacy expectations partly support the assumptions of Hypothesis 3 (i.e., that a high-efficacy cue increases respondents’ positive attitudes and efficacy expectations compared to a low-efficacy cue). Low-achieving children with ADHD, on the other hand, may have evoked respondents’ aspirations to support and engage in inclusive education. Depictions of a high chance of successful learning for children with ADHD may have contributed to a lower degree of perceived necessity and ultimately the lower efficacy of an inclusive educational setting. Such pupils may have been perceived as already being successful learners despite their ADHD, leading to the assumption that participants felt less needed or less able to promote the pupils’ learning success. However, this situation seems to be the case only for pupils with ADHD, since pupils with ID who were presented as successful learners were associated with high efficacy expectations. This finding may have been due to the cognitive nature of the latter pupils’ disability, indicating that their learning success was not perceived as the same as that of other pupils and that it continued to indicate a need for further educational intervention. In summary, the prospect of learning success described moderated the effect of the pupils’ type of disability, but not as consistently as presumed by Hypothesis 3. Since the depicted efficacy cues exhibited no other effects, support for this hypothesis is limited to the context of the ID label. Further research should examine how and which efficacy cues are most useful for reducing stigmatization and promoting the efficacy expectations of future teachers.

Student and trainee teachers’ general teaching self-efficacy emerged as an important factor with regard to shaping attitudes toward and expectations of inclusive education. As predicted by Hypothesis 4, respondents with high teaching self-efficacy were more confident and positive with respect to this topic than respondents with low teaching self-efficacy. This finding is in line with the conclusions of previous studies (Savolainen et al., 2011; Röhm et al., 2018) investigating the influence of teaching self-efficacy on attitudes toward inclusive education. However, respondents’ teaching self-efficacy did not interact with the efficacy cues depicted. Hence, Hypothesis 5, which presumed that if low-efficacy cues are salient, student and trainee teachers with high teaching self-efficacy report more positive attitudes toward and higher efficacy expectations of inclusive education as well as less social distance than student and trainee teachers with low teaching self-efficacy, must be rejected. Moreover, the observed main effect of teaching self-efficacy was also reflected in a higher-order interaction involving the pupils’ type of disability as well as respondents’ status as either students or trainees. These effect patterns additionally indicate a difference between student teachers’ and trainee teachers’ self-efficacy in an inclusive educational setting with respect to pupils with ADHD and ID, respectively, depending on their general teaching self-efficacy as a trait. In the low teaching self-efficacy condition, student teachers felt more equipped to educate pupils with ADHD than trainee teachers. When teaching self-efficacy was high, however, student teachers were more confident of their ability to educate pupils with ID than trainee teachers. This finding could suggest that trainee teachers, who have actual experience with teaching, exhibit reduced self-efficacy expectations with respect to certain teaching constellations and specific pupils. This finding is in line with the results of a recent meta-analysis (Dignath et al., 2022), which reports that “self-efficacy beliefs were found to be higher for preservice than for in-service teachers” (p. 24). Other scholars likewise emphasize the effect of teaching experience on attitudes toward inclusive education (e.g., Avramidis and Kalyva, 2007). Otherwise, student teachers may have overestimated their actual competencies. These findings deserve further investigation in future studies.

Finally, the depiction of the pupil’s sex did not make any difference on respondents’ reactions, which is in line with the findings of some researchers (e.g., Zensen and Röhm, 2021) but conflicts with those of other scholars (Burusic et al., 2012; Röhm et al., 2018). Nevertheless, there is no consistent answer to the question of whether female or male pupils are more stigmatized or rated more favorably, especially in inclusive educational settings.

Overall, the present study provides valuable insights into the influences and limitations of different causal explanations, various types of disability, and efficacy cues as well as into teacher characteristics with respect to stigma, attitudes, and expectations of inclusive education. With regard to the applicability of the mixed-blessings model (Haslam and Kvaale, 2015) to this context, the findings support only a few of the model’s original assumptions and do not indicate any disability-specific effects. Thus, several questions remain unanswered: How and which aspects, symptoms, and causes should be emphasized regarding the three exemplary types of disability referenced by this study to reduce stigmatization and promote educational efforts? Which information, in turn, should not be emphasized or should even be withheld when discussing specific pupils to prevent accidental stigmatization? To what extent is the observed positive effect of a psychosocial causal explanation transferable to other types of disability?

In addition to the effects of the attributed causes, the disability labels used in this study evoked complex patterns of reaction from respondents depending on other influencing factors. With regard to the assumptions drawn from labeling theory (Link and Phelan, 2001), no uniform stereotypical reactions emerged with respect to any of the disability labels. Respondents’ reactions were instead influenced by additional contextual information that either affirmed (e.g., pupils with ID and low chance of learning success) or contradicted (e.g., pupils with ID and high chance of learning success) stereotypical expectations. In addition, teaching self-efficacy played an important role with regard to the effects of the case vignette and produced the largest effect sizes. An integrative model of stigmatization and attitudes in the context of inclusive education, such as the adapted mixed-blessings model, should integrate the impact of student and trainee teachers’ individual dispositions and experiences to account for the characteristics of both stigmatizers and the stigmatized (*cf.* Möhring et al., 2021).

### Limitations

The current study examined the factors that shape teachers’ attitudes toward inclusive education and social distance toward children with a disability in depth. The interpretation and generalizability of the results is to some extent limited to the context of the German education system and its implementation of inclusive education. Since we collected only explicit self-reported data, a certain degree of social desirability bias must be expected. Due to our use of an online experiment, issues such as a high dropout rate and self-selection bias may further limit the generalizability of our results (Reips, 2002), although the required sample size of approximately *n* = 632 that was estimated a priori was almost met. Although the high dropout rate is not uncommon for online studies (*cf.* Daikeler et al., 2020) the results from the MCAR test showed that participants did not abort the questionnaire at random (especially at the beginning or directly after the stimulus). This could indicate that participants that are generally less motivated, interested, or even opposed to the topic of inclusive education were to a lesser extent included into our final analyses. Future studies should aim to also include those views by, for example, adopting more research-economically scales, being sensitive to reactance, and providing incentives to guide participants attention und motivation throughout the questionnaire. Since we targeted a rather specific sample, the self-selection bias of student and trainee teachers was limited to their sex and age, characteristics which exhibited similar distributions to those found in previous studies (Röhm et al., 2018; Zensen and Röhm, 2021). Regarding respondents’ sex, the present sample reflects the general demographics of the population, particularly that of primary and special education teachers (UNESCO, 2019). However, respondents’ sex was not included as a factor in the analysis to prevent unequal distributions among the experimental and quasi-experimental factors. Thus, future studies should focus in more detail on the attitudes of male students and trainee teachers, who are known to be more stigmatizing than females (Kaushik et al., 2016). In addition, researchers should also aim to examine the possible effects of student and trainee teachers’ previous experiences with pupils with disability on stigma-related attitudes in more detail. This factor could not be controlled for in the present study due to the relatively low number of student and trainee teachers with special educational teaching backgrounds. The present study further aimed to limit (and control for) the role of contextual factors such as teaching conditions (e.g., overcrowding) and classroom management (*cf.* Blotnicky-Gallant et al., 2015) by refraining from describing them as much as possible and therefore ensuring that their salience to the respondents remained low. However, the experimental manipulation of the pupil’s depicted need for support and prospects of learning success addressed some of these issues, which, in addition to the type of disability in question, may play an important role regarding teachers’ perceptions of and interactions with the pupil.

Since the analyses conducted for this study were strictly controlled with regard to type 1 errors and only MANOVA effects at the 1% level of significance were included into further analyses, effects with 0.01 < *p* < 0.05 were excluded, which may also have provided additional insights. Further investigations may employ a more powerful approach to examine the impact of each individual manipulation as well as the possible associated long-term effects. Regarding the operationalization of the constructs related to the mixed-blessings model, the measures applied with regard to prognostic pessimism yielded the hypothesized effects. Future studies should also employ scales that measure factors other than positive attitude dimensions. In addition, there was no direct operationalization of attributions of uncontrollability or essentialist beliefs as mediators as performed in, for instance, the recent study by Dittrich et al. (2021). Thus, interpretations of attributed uncontrollability or, in particular, essentialism relied on the link between the depicted cause and its effects on stigmatization and prognostic pessimism. Furthermore, the wording of the items employed to measure teaching self-efficacy may have biased respondents’ responses regarding stigmatization due to statements such as “I know that I am able to teach test-relevant content even to problematic pupils.” Additionally, in contrast to previous studies (Röhm et al., 2018; Zensen and Röhm, 2021), the social distance scale used in this study yielded only a comparably low reliability. This scale was also related only to personal social distance toward children with a disability in general instead of to teachers’ social distance toward students with a disability. Hence, future studies should aim to operationalize all aspects of the mixed-blessings model in a more detailed, more reliable, and less biased way.

### Conclusion

To prevent professional or institutional stigmatization, teacher education and training as well as communication regarding pupils with disabilities require a high degree of sensitivity to disability-specific and efficacy-related cues that can, in one arrangement, promote the inclusion of a certain group but may, in other contexts, lead to unintended reactions or expectations. Supporting future teachers’ self-efficacy deserves greater attention with regard to the task of establishing a more inclusive education system, as do appropriate forms of teacher training that include essentials from anti-stigma communication.

## Data availability statement

The raw data supporting the conclusions of this article will be made available by the authors, without undue reservation.

## Ethics statement

Ethical review and approval was not required for the study on human participants in accordance with the local legislation and institutional requirements. Written informed consent for participation was not required for this study in accordance with the national legislation and the institutional requirements.

## Author contributions

AR conceptualized the study, conducted the data analysis, and wrote the first draft. MG, JZ-M, and AR organized and conducted the sample recruitment with contributions. MG, MM, JZ-M, CN, and MH contributed to the design, stimulus material, and operationalization of constructs. All authors contributed to the article and approved the submitted version.

## Conflict of interest

The authors declare that the research was conducted in the absence of any commercial or financial relationships that could be construed as a potential conflict of interest.

## Publisher’s note

All claims expressed in this article are solely those of the authors and do not necessarily represent those of their affiliated organizations, or those of the publisher, the editors and the reviewers. Any product that may be evaluated in this article, or claim that may be made by its manufacturer, is not guaranteed or endorsed by the publisher.
